# Independent effects of mental fatigue and drop height on drop jump performance in American football athletes: an exploration of central-peripheral interaction

**DOI:** 10.7717/peerj.20574

**Published:** 2026-01-13

**Authors:** Zilong Wang, Ziqi Feng, Mengya Lu, Jing Sun, Tao Liu, Qiuxia Zhang, Xiangdong Wang

**Affiliations:** 1 School of Physical Education, Jimei University, Xiamen, China; 2 School of Public Foundation, Dalian University of Technology, Panjin, China; 3 School of Physical Education, Soochow University, Suzhou, China; 4 Department of Teaching and Research, Dalian University of Technology, Panjin, China

**Keywords:** Mental fatigue, Drop jump, Athletic performance, American football, Drop height

## Abstract

**Background:**

This study aimed to investigate the effects of mental fatigue (MF) and different drop heights on the athletic performance of American football players executing the drop jump (DJ) movement.

**Methods:**

Twelve male American football athletes were selected as subjects. The Vicon infrared three-dimensional motion capture and analysis system, Kistler three-dimensional force platform, and other instruments were used. MF was induced through a Stroop task, and the DJ performance of the subjects was tested at drop heights of 30 cm, 40 cm, and 50 cm. A 2 × 3 repeated measures analysis of variance was employed.

**Results:**

Regarding different jump heights, both under MF and baseline conditions, 40 cm and 50 cm heights resulted in lower jump heights compared to the 30 cm height (*p* = 0.002, *p* = 0.008); in terms of the rate of force development (RFD) metric, both under MF and baseline conditions, 40 cm and 50 cm heights showed lower RFD compared to the 30 cm height (*p* < 0.001); in the average power output metric, compared to the baseline, MF resulted in lower average power output across different drop heights (*p* < 0.001); in the reactive strength index (RSI) metric, compared to the baseline, MF resulted in lower RSI across different drop heights (*p* = 0.001), and compared to the 30 cm height, 40 cm and 50 cm heights showed lower RSI (*p* = 0.004, *p* = 0.008); in the Reactive Strength Ratio (RSR) metric, compared to the baseline, MF resulted in lower RSR across different drop heights (*p* = 0.001); in the K_leg_ metric, compared to the 30 cm height, 40 cm and 50 cm heights showed higher K_leg_ (*p* = 0.001, *p* = 0.008).

**Conclusions:**

Under MF conditions, athletes’ performance in average power output, RSI, and RSR declined, suggesting a reduction in central nervous system efficiency. Additionally, increasing the drop height to 40 cm or above significantly reduced jump height and RFD, and increased K_leg_. However, no interactive effects between MF and drop height were observed.

## Introduction

American football is a globally popular team sport, renowned for its high-intensity physical confrontations, complex tactical strategies, and the extreme demands it imposes on athletes’ comprehensive physical fitness (Hoffman, 2008; Liu et al., 2020; Robineau et al., 2017). In competitive sports, the performance of American football players is influenced by numerous factors, including physical and psychological elements. This sport demands not only exceptional physical qualities from athletes but also rapid decision-making abilities and tactical comprehension. Consequently, mental fatigue (MF) may significantly impact athletes’ on-field performance (Mariano, Martin & Mara, 2023). Particularly in the critical moments before a game, prolonged mental concentration and decision-making pressures can degrade cognitive functions, thereby impairing reaction speed, judgment accuracy, and tactical execution capabilities (Schampheleer & Roelands, 2024; Staiano et al., 2024).

MF typically refers to a reduction in the brain’s information processing capacity following prolonged cognitive tasks, manifesting exhaustion and a lack of energy (Marcora, Staiano & Manning, 2009). Furthermore, as modern life accelerates and technological products become more prevalent, athletes encounter increased mental exertion outside of training and competition. For example, the frequent use of smartphones, electronic games, and sleep deprivation can all trigger MF, and recovering from this state of fatigue is challenging within a short timeframe (Filipas et al., 2021; Thompson et al., 2019; Thompson et al., 2020; Smith et al., 2019). Existing evidence demonstrates that MF can impair decision-making and technical skill execution in team invasion sports like soccer and basketball (Gantois et al., 2020; Moreira et al., 2018; Smith et al., 2016). Although these sports have different skill requirements compared to American football, they share notable similarities in match demands, such as high-intensity intermittent exercise and the need for sustained attention to the surrounding environment and teammates. These similarities imply that American football athletes might also be vulnerable to the negative effects of MF on performance. Mariano, Martin & Mara (2023) identified a significant negative correlation between MF and technical performance in a survey of 20 elite male rugby athletes, underscoring the importance of assessing MF in rugby athletes.

The drop jump (DJ) is a crucial test for evaluating lower limb explosive power and neuromuscular efficiency, with its execution precision directly reflecting an athlete’s competitive state (Leukel et al., 2008; Furuhashi et al., 2021; Yang et al., 2024). Within sports science, the DJ test is widely utilized, as it effectively simulates the stretch-shortening cycle (SSC) in actual sports movements and efficiently assesses athletes’ biomechanical characteristics and reactive strength in athletic performance (Di Giminiani & Petricola, 2016; Matic et al., 2015; Horita et al., 2002). Mrdakovic et al. (2008) and Byrne et al. (2010) investigated the effects of DJs at various drop heights (20 cm to 80 cm) on athletes’ lower limb muscle activity and ground reaction forces, uncovering differences in biomechanical efficiency across drop heights and their substantial influence on athletic performance. Nevertheless, in practical training, DJs at drop heights of 30, 40, and 50 cm have become popular training tools for coaches and athletes due to their ease of use and safety. These heights serve as a practical method for assessing athletes’ explosive power and neuromuscular coordination (Peng, 2011; Wang et al., 2021). For American football athletes, DJ training at these drop heights enhances explosive power and agility while effectively mirroring the game’s actual movement demands.

While extensive research exists on the effects of varying drop heights on athletes’ DJ performance (Mrdakovic et al., 2008; Byrne et al., 2010; Peng, 2011; Wang et al., 2021; Sotiropoulos et al., 2023; Ramirez-Campillo et al., 2022), limited studies have examined how MF influences the performance of athletes in high-intensity, complex decision-making sports such as American football during DJ execution. Furthermore, the impact of MF on DJs at different drop heights remains underexplored. Previous research has demonstrated that MF can impair various aspects of physical performance. For instance, Lima-Junior et al. (2024) found that MF induced by smartphone use negatively affected inhibitory control, though it did not significantly impact countermovement jump performance in trained individuals. Similarly, Queiros et al. (2021) observed that MF reduced training volume in resistance exercises, indicating a potential influence on athletic performance. On the other hand, Díaz-García et al. (2024) highlighted that brain endurance training could mitigate the detrimental effects of MF on resistance exercise performance. Consequently, this study employs DJs at 30, 40, and 50 cm drop heights to investigate how MF affects the DJ performance of American Football athletes at these frequently used heights, offering novel insights into athletic performance under high-pressure conditions.

Given this context, this study aims to investigate how MF affects the athletic performance of American football athletes performing DJ movements at different drop heights. The study seeks to provide experimental data and theoretical insights regarding the effects of MF on DJ performance in American football athletes. Drawing on prior research, the following hypotheses are proposed: (1) MF has a lesser impact on low-height DJ than on high-height DJ; (2) MF does not significantly affect the jump height of American football athletes at different drop heights during DJ; (3) different drop heights significantly impact the execution of DJ movements by American football athletes.

## Materials & Methods

### Study design

This study employed a within-subjects repeated-measures design to investigate the independent effects of MF and different drop heights on the DJ performance of American football athletes. The design was chosen to specifically address the knowledge gap regarding how MF impacts the DJ performance of athletes in high-intensity, complex decision-making sports like American Football at frequently used drop heights. By incorporating an orientation phase for participant familiarization and a practice session to ensure understanding of experimental procedures, the study aimed to provide precise and reliable data. It included two main conditions: a MF intervention induced by a 45-minute Stroop task, and a control condition without MF intervention. DJ tests were conducted at varying drop heights both before and after the intervention to assess the influence of MF on athletic performance, thereby offering valuable insights for the training management, fatigue recovery, and competitive state optimization of American football athletes.

### Participants

G*Power 3.1.9 software was utilized for sample size estimation. Based on prior studies (Faul et al., 2007; Brysbaert, 2019; Griffin et al., 2021) and the statistical methods of this research, parameters including power (1−β) of 0.80, Type I error rate (α) of 0.05, and effect size (f) of 0.4 were set. Consequently, a minimum required sample size of 10 participants was calculated. Ultimately, 12 male athletes from the Soochow University American football team were recruited. Their average age was 22.2 ± 1.9 years, height 181.4 ± 3.1 cm, body mass 80.1 ± 8.5 kg, and training experience 4.8 ± 1.5 years. All participants were key players in the China Collegiate American football League championship team, with extensive competitive experience at the national level. Their current training routine includes three to five sessions per week, encompassing technical drills, tactical practices, and physical conditioning, each lasting 2–3 h. Additionally, all subjects had no prior history of professional training in other sports, ensuring specialization in American football. They were thoroughly informed about the test procedures and potential risks, and provided written consent. The study was approved by the Ethics Committee of Soochow University (Approval No. SUDA20240626H03). The inclusion criteria were established to ensure the selection of a homogeneous sample with high physical fitness, free from lower limb joint or neuromuscular diseases or injuries in the past six months, similar morphological characteristics, no intense exercise 24 h before testing, no caffeine intake, abstinence from alcohol for at least one week before testing, and a stable psychological state without severe disorders or excessive stress. These criteria were designed to minimize variability and potential confounding factors, ensuring the sample’s representativeness and suitability for the study.

### Experimental equipment

#### Vicon infrared 3D motion capture and analysis system

The system consisted of eight infrared cameras (Model: MX13, Vicon Motion Systems, Oxford, UK), MX Net, MX Control, a PC workstation, calibration kits, and standard accessories. It operated at a sampling frequency of 100 Hz and used the Plug in Gait Full-Body model in Vicon Nexus software for motion tracking and data acquisition.

#### Kistler 3D force platform

Two Kistler 3D force platforms (90 cm × 60 cm × 10 cm, Model: 9281EA, Kistler Instruments, Winterthur, Switzerland) were embedded in the ground within the motion capture system’s infrared camera range. They operated at a sampling frequency of 1000 Hz and were synchronized with the Vicon system *via* an analog-to-digital converter for data collection.

### Study design and procedure

**Figure 1:**
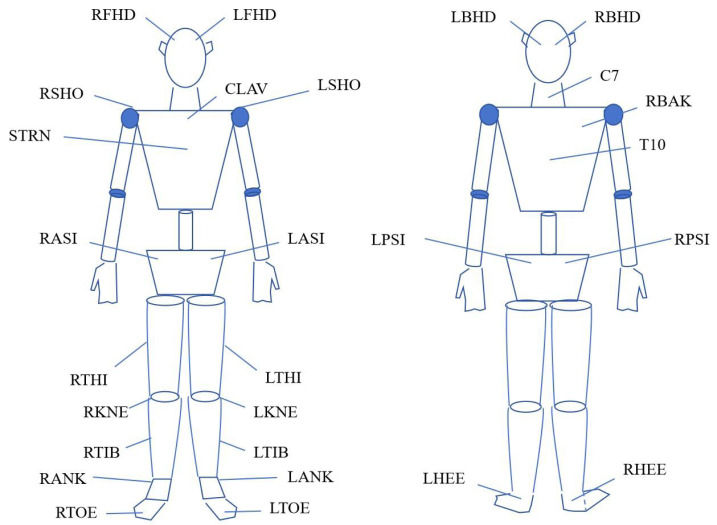
Schematic diagram of marker attachment. RFHD, Right Front Head (Temple); LFHD, Left Front Head (Temple); RSHO, Right Shoulder (Acromion); CLAV, Clavicle (Suprasternal Notch); LSHO, Left Shoulder (Acromion); STRN, Sternum (Xiphoid Process); RASI, Right Anterior Superior Iliac Spine; LASI, Left Anterior Superior Iliac Spine; RTHI, Right Thigh; LTHI, Left Thigh; RKNE, Right Knee (Lateral Epicondyle); LKNE, Left Knee (Lateral Epicondyle); RTIB, Right Tibia (Shank); LTIB, Left Tibia (Shank); RANK, Right Ankle (Lateral Malleolus); LANK, Left Ankle (Lateral Malleolus); RTOE, Right Toe (2nd Metatarsal Head); LTOE, Left Toe (2nd Metatarsal Head); LBHD, Left Back Head; RBHD, Right Back Head; RBAK, Right Back (Right Scapula); LPSI, Left Posterior Superior Iliac Spine; RPSI, Right Posterior Superior Iliac Spine; LHEE, Left Heel (Calcaneus); RHEE, Right Heel (Calcaneus).

Prior to the commencement of each experimental session, participants underwent a familiarization phase that included practice trials of the DJ movement at all tested heights (30 cm, 40 cm, 50 cm) to ensure understanding and consistency. Participants were required to wear tight-fitting shorts and athletic shoes provided by the laboratory. All experiments were conducted in a controlled laboratory environment with temperature maintained at 22–24 °C and relative humidity at 50–60%. Following the completion of warm-up activities, 27 markers with a diameter of 14 millimeters were affixed to the participants’ body surfaces according to the full-body model protocol (Fig. 1). To ensure the accuracy of the experiment, the application of all markers was performed by the same experienced technician who is a PhD holder from Soochow University and a laboratory technician at the Sports Biomechanics Laboratory of Soochow University, with over 10 years of experience in motion capture studies. The equipment used included the Vicon infrared 3D motion capture system and Kistler 3D force platforms, as detailed in ‘Experimental equipment’. Participants were instructed to execute each DJ with maximal effort, focusing on minimizing ground contact time and maximizing jump height. Data collection was synchronized between the motion capture and force platforms *via* an analog-to-digital converter, and raw data were processed using Visual 3D software with filtering parameters as described. All experimental sessions were conducted during the non-competitive season, specifically within the one-week transition phase following the competitive season. To minimize circadian rhythm effects, all sessions were scheduled between 15:00 and 19:00, as recommended by Wang et al. (2025), to ensure task-induced fatigue predominated over physiological fluctuations.

Before the initiation of testing, a questionnaire was employed to assess the compliance of the participants. Subsequently, The National Aeronautics and Space Administration Task Load Index (NASA-TLX) Subjective Workload Scale was used to measure the participants’ workload levels before the commencement of the test. After the completion of the MF task, the NASA-TLX and other indicators were measured again to assess the participants’ workload levels (Wang et al., 2025). Following this, the participants performed an additional approximately 2-minute warm-up before immediately proceeding with the corresponding tests.

**Figure 2:**
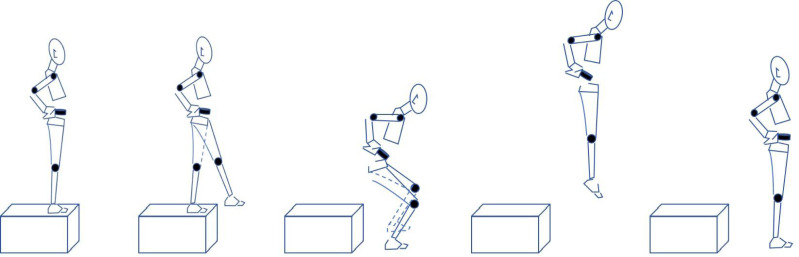
Action diagram.

Participants stood on a plyometric box with hands on hips, awaiting instructions. Upon command, they stepped forward, fell freely, and performed a full DJ movement sequence: “landing-contact-jump-off-airborne-landing”. The movement included contact and airborne phases, with the contact phase further divided into braking and push-off phases (Fig. 2). Athletes maintained balance and minimized ground contact time to maximize jump height. Each DJ test had a 30-second recovery interval, and three successful tests were collected for each drop height. The maximum jump height from the three trials was used for analysis.

### Experimental protocol

The MF intervention involved participants engaging in a 45-minute Stroop task, designed based on the studies by Lopes et al. (2020) and Badin et al. (2016) and proven effective in inducing MF (Mangin et al., 2022). On a computer screen, the Chinese characters for “red”, “green”, “blue”, and “yellow” appeared one at a time in random order, each displayed in one of the four colors. The probability of the character’s color not matching its meaning was set at 50%, and participants pressed a button based on the character’s color. Each character was displayed for 1,000 ms, followed by a 1,000 ms blank screen interval before the next character appeared. Participants completed a total of 1,350 judgment reactions during the task. They were instructed to respond as quickly and accurately as possible. If a response was incorrect or no response was made within 1,500 ms, the system emitted a beep to encourage greater focus. The experiment took place in a separate, quiet room, with E-prime 3.0 software managing task operations. Two staff members supervised the process to ensure smooth progress and participant concentration. The parameters of the Stroop task, including character presentation time and the probability of color—meaning mismatch, were determined through reference to prior studies and pilot experiments. These parameters were optimized to impose sufficient cognitive load on participants, ensuring the effective induction of MF and providing a reliable foundation for subsequent assessments of its impact on athletic performance.

### Data collection and analysis

Raw Vicon data were analyzed in Visual 3D software (C-Motion, Germantown, MD, USA) for kinematic and inverse dynamics analysis. A 4th-order low-pass Butterworth filter smoothed the three-dimensional coordinates (10 Hz) and force platform data (50 Hz). Takeoff and landing were defined as when the vertical ground reaction force (vGRF) was less than or exceeded 10 N (Decker et al., 2003; Slater et al., 2015; Wang et al., 2024).

The main observational indices in this study were:

(1) The maximum takeoff height normalized by body height (BH), calculated using the waist point method (Wan et al., 2017). This index reflects the athlete’s vertical jumping ability during DJ, serving as a key measure of lower limb explosive power and athletic performance.

(2) The peak vGRF during the push-off phase (vGRF_peak_, N). It represents the maximum vertical ground reaction force generated by the athlete during the push-off phase, reflecting the athlete’s lower limb strength and ability to exert force on the ground

(3) The rate of force development (RFD, N/s) = vGRF_peak_/K_t_, where K_t_ is the time required to reach the peak force during the push-off phase (Molina & Denadai, 2012). RFD reflects the speed and efficiency with which muscles develop force, and is an important indicator of rapid strength and neuromuscular coordination. It helps assess the athlete’s ability to exert force quickly during the push - off phase of DJ.

(4) Average power output (W/kg) = (g^2^ × T_f_ × T_t_)/(4 × T_c_); Reactive Strength Index (RSI, m/s) = H_f_/T_c_; Reactive Strength Ratio (RSR) = T_f_/T_c_, where g is the acceleration due to gravity (9.8 N/kg), T_f_ is the time in the air after the push-off phase ends, H_f_ is the takeoff height, T_c_ is the contact time from the first moment of foot contact to the moment of takeoff, and T_t_ = T_f_ + T_c_ (Bosco, Luhtanen & Komi, 1983; Healy, Kenny & Harrison, 2018; Healy, Kenny & Harrison, 2016). These indices comprehensively evaluate the athlete’s power output, reactive strength, and movement efficiency during DJ, providing a detailed assessment of athletic performance.

(5) Lower limb stiffness (K_leg_, BW/m) = Fz_max_/ΔL, where Fz_max_ represents the maximum vertical ground reaction force normalized by body weight during the braking phase, and ΔL represents the maximum change in lower limb length, *i.e.,* the vertical displacement change of the hip joint center from the moment of contact to the completion of braking (You & Huang, 2022). K_leg_ reflects the stiffness characteristics of the lower limbs during landing, which is related to the athlete’s ability to absorb impact force and store elastic energy, and has a significant impact on DJ performance and injury prevention.

### Statistical analysis

Data were analyzed using SPSS 26 (IBM Corp., Armonk, NY, USA). Descriptive statistics are reported as mean ± standard deviation (M ± SD). The Shapiro–Wilk test assessed normality, and Levene’s test assessed homogeneity of variance. Paired-samples t-tests compared NASA-TLX scores pre- and post-intervention. A 2 × 3 (condition (baseline, MF) × height (30, 40, 50 cm)) repeated-measures ANOVA was conducted. Mauchly’s test assessed sphericity; when violated, Greenhouse–Geisser corrections were applied. Statistical significance was set at α = 0.05, with Bonferroni-adjusted *post hoc* tests.

## Results

### MF intervention outcomes

**Figure 3:**
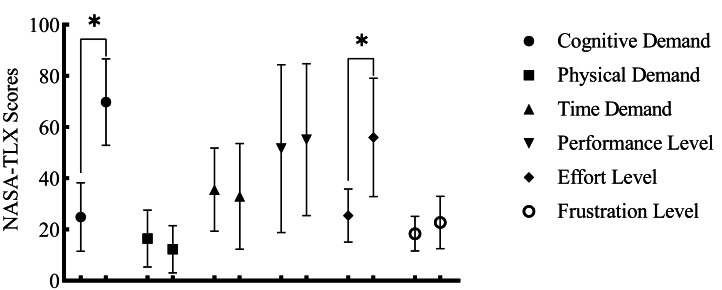
NASA-TLX Scores before and after the MF. *p < 0.05.

In the MF protocol, there was a significant increase in the cognitive demand (*p* < 0.001) and effort expenditure (*p* = 0.002) of the participants, confirming the successful induction of MF (Fig. 3).

### Effects of MF on jump height and vGRF_peak_ during push-off phase

**Figure 4:**
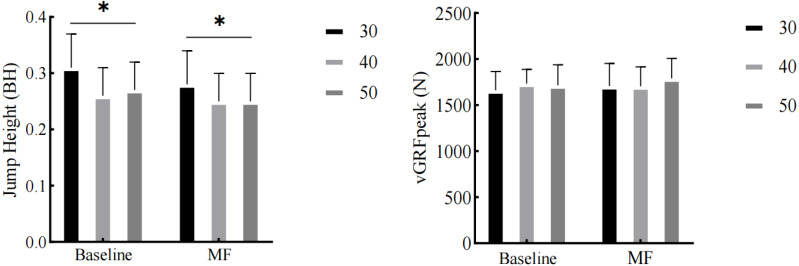
The impact of MF on jump height and the vGRF_peak_. *p < 0:05.

A significant main effect of drop height on jump height was observed (*p* < 0.001). *Post-hoc* comparisons revealed that, under both MF and baseline conditions, the heights of 40 cm and 50 cm resulted in lower jump heights compared to the 30 cm height (*p* = 0.002, *p* = 0.008). No statistically significant differences were observed for the vGRF_peak_ measure (*p* > 0.05) (Fig. 4).

### Effects of MF on RFD, average power output, RSI, RSR, and K_leg_

A significant main effect of drop height on the RFD measure was observed (*p* < 0.001). *Post-hoc* comparisons revealed that, under both MF and baseline conditions, the 40 cm and 50 cm heights resulted in lower RFD compared to the 30 cm height (*p* < 0.001). A main effect of MF on average power output was observed, with lower average power output observed across different drop heights after MF compared to baseline (*p* < 0.001). For the RSI measure, main effects of MF and drop height were observed (*p* = 0.001 for both). *Post-hoc* comparisons indicated that, compared to baseline, lower RSI was observed after MF across different drop heights (*p* = 0.001), and lower RSI was observed at 40 cm and 50 cm heights compared to the 30 cm height (*p* = 0.004 and *p* = 0.008, respectively). A main effect of MF on RSR was observed, with lower RSR observed across different drop heights after MF compared to baseline (*p* = 0.001). A significant main effect of drop height on K_leg_ was observed (*p* = 0.002). *Post-hoc* comparisons revealed that, compared to the 30 cm height, higher K_leg_ was observed at 40 cm and 50 cm heights (*p* = 0.001 and *p* = 0.008, respectively) (Table 1).

**Table 1:** The impact of MF on average power output, RFD, RSI, RSR, and K^leg^. *p < 0:05.

**Variables**	**Baseline**	**MF**	**Main effect**		**Interaction effect**
			**MF**	**Height**	**MF × Height**
RFD (N/s)					
30 cm	9927.54 ± 1682.56	8590.23 ± 1433.50	*p*= 0.057	***p*** < **0.001**^∗^	*p*= 0.303
40 cm	7246.60 ± 1752.35	6036.76 ± 1629.16	*F* = 4.537	*F* = 26.070	*F* = 1.261
50 cm	6885.17 ± 1767.35	6528.97 ± 1574.66	*Eta*^2^= 0.292	*Eta*^2^= 0.703	*Eta*^2^= 0.103
Average power output (W/kg)					
30 cm	29.94 ± 5.21	25.19 ± 3.86	***p*** < **0.001**^∗^	*p* = 0.189	*p*= 0.118
40 cm	27.84 ± 5.25	24.78 ± 3.39	*F* = 24.249	*F* = 1.797	*F* = 2.361
50 cm	27.86 ± 5.79	24.45 ± 4.36	*Eta*^2^= 0.688	*Eta*^2^= 0.140	*Eta*^2^= 0.177
RSI (m/s)					
30 cm	1.10 ± 0.29	0.85 ± 0.19	***p***=**0.001**^∗^	***p*=** **0.001** ^∗^	*p*= 0.322
40 cm	0.88 ± 0.24	0.74 ± 0.18	*F* = 20.462	*F* = 14.208	*F* = 1.131
50 cm	0.87 ± 0.26	0.73 ± 0.18	*Eta*^2^= 0.650	*Eta*^2^= 0.564	*Eta*^2^= 0.093
RSR					
30 cm	1.07 ± 0.23	0.87 ± 0.16	***p*=** **0.001** ^∗^	*p* = 0.511	*p*= 0.256
40 cm	1.04 ± 0.21	0.89 ± 0.15	*F* = 18.470	*F* = 0.692	*F* = 1.451
50 cm	1.02 ± 0.23	0.87 ± 0.15	*Eta*^2^= 0.627	*Eta*^2^= 0.059	*Eta*^2^= 0.117
K_leg_ (BW/m)					
30 cm	40.02 ± 16.87	50.57 ± 8.96	*p* = 0.499	***p*=** **0.002** ^∗^	*p* =0.897
40 cm	56.22 ± 13.84	61.72 ± 10.03	*F* = 0.490	*F* = 11.782	*F* = 0.109
50 cm	63.19 ± 19.99	66.26 ± 19.21	*Eta*^2^= 0.043	*Eta*^2^= 0.517	*Eta*^2^= 0.010

## Discussion

The study results indicate that MF significantly affects the performance of American football athletes in executing DJs, particularly in terms of average power output, RSI and RSR, but not in jump height, vGRF_peak_, RFD, or K_leg_. Additionally, no interactive effects were found between MF and drop height, suggesting that MF does not differentially affect DJs at various drop heights tested in this study, contradicting our initial hypothesis. The impact of MF primarily lies in cognitive function and decision-making processes. Although MF does not directly lead to muscle fatigue, its negative effects on cognitive function and decision-making may indirectly affect athletic performance (Smith et al., 2018; Smith et al., 2019). Therefore, the decline in average power output, RSI, and RSR under MF may be associated with reduced efficiency of the central nervous system. This is consistent with the findings of Brown et al. (2020), who concluded that MF impairs physical performance and thus affects movement execution. Furthermore, Davidow et al. (2023) found that MF significantly reduced tackling technique proficiency in amateur rugby union players, suggesting that impaired motor coordination under MF may stem from deficits in executive functions and suboptimal neural signaling. Therefore, in high-pressure environments, athletes’ technical execution and decision-making abilities may be negatively affected by MF.

It is noteworthy that although MF had a certain negative impact on average power output, RSI, and RSR during the execution of DJs, it did not significantly affect jump height and RFD, which aligns with our hypothesis 2. Similar to previous findings by Weerakkody et al. (2021), after a 30-minute Stroop test intervention on 25 amateur Australian football athletes, MF did not affect the jump height during either standing vertical jumps or running vertical jumps. The resistance of RFD to MF might be attributed to its dependence on intrinsic muscle–tendon unit properties and well-learned movement patterns, which could be less susceptible to cognitive fatigue compared to more complex, power-dependent outputs requiring optimal central drive and coordination. Considering that American football is a sport with high cognitive function demands, athletes need to maintain a high level of alertness and concentration during matches and training to capture and process information from the rapidly changing field environment (MacDonald & Minahan, 2016). This sustained cognitive load often induces MF, and frequent competitions and training further increase the frequency with which athletes are in a state of MF (Abbott et al., 2020). This frequent exposure necessitates that athletes develop effective psychological and behavioral coping strategies to manage MF. Research indicates that experienced athletes often demonstrate superior abilities in regulating cognitive resources and maintaining performance under pressure (Russell et al., 2020; Martin et al., 2016). This suggests that extensive competition experience may make athletes more efficient in managing cognitive resources and coping with psychological stress, thereby maintaining better athletic performance under high-pressure competitive conditions. Therefore, the results of this study indicate that athletes may effectively manage stress and fatigue by developing psychological resilience and adopting effective coping strategies, thus maintaining their athletic performance to a certain extent in high-pressure environments. This finding emphasizes the importance of psychological skill training in athletes’ training programs and its potential to enhance athletes’ performance under pressure.

In this study, we observed that drop height significantly affected the performance of American football athletes in executing DJs. Specifically, compared with the 30 cm drop height, the 40 cm and 50 cm heights resulted in lower jump heights and RFD, as well as lower RSI under both baseline and MF conditions, while higher values were observed for the K_leg_ indicator. These observations reveal the significant impact of drop height on athletes’ neuromuscular coordination and energy conversion efficiency, consistent with our hypothesis 3. The study results are similar to those of Bobbert, Huijing & van Ingen Schenau (1987), who emphasized the impact of drop height on muscle activation regulation and athletic performance by comparing the differences in DJ performance at drop heights of 20–60 cm. This further supports our conclusion and highlights the importance of considering drop height in training for athlete performance and health.

From a biomechanical perspective, increasing the drop height requires athletes to absorb more impact force during the landing phase, which may affect their subsequent takeoff ability. This phenomenon is consistent with the research of Lesinski et al. (2018), who suggested that higher drop heights may prompt athletes to adopt more conservative movement strategies to reduce the risk of injury. Additionally, the higher K_leg_ indicates that athletes may increase muscle pre-activation to improve the absorption of impact force, which to some extent maintains their jumping performance. This is consistent with the research results of Peng (2011), who pointed out that jumping from a height exceeding 40 cm offers no advantage in SSC power output and K_leg_ and suggested avoiding DJ from heights exceeding 60 cm, as it lacks biomechanical efficiency and may increase the risk of injury. This is also consistent with previous studies on the adaptive changes of the muscle–tendon complex in response to greater impact forces, which may involve changes in muscle fiber recruitment patterns, motor unit activation sequences, and intramuscular coordination and synchronization (Dyhre-Poulsen, Simonsen & Voigt, 1991; Chen et al., 2022).

Furthermore, the lower jump height and RFD, as well as the lower RSI, suggest that at higher drop heights, athletes may rely more on muscle strength rather than speed to complete the movement, which may be a natural response to reduce the impact force upon ground contact. This strategic change is consistent with previous studies observing muscle activity patterns, where athletes adjust their muscle activation patterns to adapt to different movement demands during high-intensity jumping actions (Lesinski et al., 2018). However, these findings are inconsistent with the previous research of Matic et al. (2015), who found that the optimal load height for athletes with high lower limb strength was 50 cm, and for those with low lower limb strength was 35 cm through experiments with 8 drop heights. In addition, Di Giminiani & Petricola (2016) found that the drop height for male athletes to achieve the maximum average power output in DJ was around 40 cm by comparing the power output at drop heights of 20 cm to 60 cm. These differences may stem from the characteristics of the subject groups; Matic et al. and Giminiani et al. studied male students rather than professional athletes, and not unified in a single sport, which may have affected the universality of the results. Determining the optimal drop height in DJ training is a complex issue influenced by various variables, including experimental design, data collection and analysis strategies, and athletes’ lower limb strength capabilities (Zang et al., 2019). The data from this study suggest that for American football athletes, a 30 cm drop height may provide a more appropriate training load, as American football athletes often need to perform multidirectional jumps and change movements with rapid reaction characteristics in daily training and matches, rather than a single maximum force output. The explosive power and coordination required by the 30 cm drop height may be closer to the actual needs of American Football, making this height an effective training load to improve their competitive performance.

In summary, this study reveals that both MF and drop height are key factors affecting the lower limb movement performance of American football athletes when executing DJs. These findings are significant for developing targeted training programs, especially considering individual differences and training goals of athletes. Future research should further explore the impact of different drop heights on athletes’ technical and physiological adaptations to better understand the mechanisms behind these phenomena and provide more personalized training recommendations for athletes. By gaining a deeper understanding of the impact of drop height on athletic performance, more scientific training guidance can be provided for athletes, optimizing training effects and reducing the risk of sports injuries.

This study has several limitations. First, although the sample size (*n* = 12) met the minimum requirement based on our a priori power calculation, it remained relatively small and consisted exclusively of male collegiate American football athletes. This may limit the generalizability of the findings to athletes of different genders, age groups, skill levels, and competitive backgrounds. Future research should therefore recruit larger and more diverse samples to enhance statistical power and external validity. Second, the conclusions were based on controlled laboratory conditions, with drop heights restricted to 30–50 cm, leaving uncertainty as to whether the findings can be extended to real competitive environments or to other drop heights. Third, mental fatigue was assessed primarily through a subjective workload scale, and the control condition employed a no-intervention design. Future studies could incorporate physiological or behavioral indicators of mental fatigue and utilize a low-cognitive-demand active control task to improve internal validity and the precision of interpretations regarding fatigue-related effects.

## Conclusions

This study investigated the impact of MF and drop height on the DJ performance of American football athletes. Under conditions of MF, a decline in average power output, RSI, and RSR was observed in athletes, suggesting reduced efficiency in central nervous system function. Additionally, increasing the drop height to 40 cm or above significantly reduced jump height and RFD, and increased K_leg_, indicating that athletes may adopt more conservative movement strategies to reduce the risk of injury. However, no interactive effects between MF and drop height were observed. These findings emphasize the importance of considering the impact of MF and drop height on athlete performance in sports that demand high intensity and complex decision-making. Future training and research should focus on how to mitigate the negative effects of MF through strategic training interventions and optimize drop height to enhance athletic performance and reduce the risk of injury.

## Supplemental Information

10.7717/peerj.20574/supp-1 Supplemental Information 1 Raw data files for each set of experiments

## Additional Information and Declarations

### Data Availability

The following information was supplied regarding data availability: All raw data from the experiments are available in the Supplemental File.
